# Supplementary material for: Proteome profile of peripheral myelin in healthy mice and in a neuropathy model
Source: eLife. 2020 Mar 4;9:e51406. doi: 10.7554/eLife.51406 (PMC7056269; doi:10.7554/eLife.51406)
Supplement: Transparent reporting form [file elife-51406-transrepform.pdf]

1st Floor
24 Hills Road
Cambridge CB2 1JP, UK

P 01223 855340
W elifesciences.org
T @elife

eLife's transparent reporting form

We encourage authors to provide detailed information *within their submission* to facilitate the interpretation and replication of experiments. Authors can upload supporting documentation to indicate the use of appropriate reporting guidelines for health-related research (see EQUATOR Network), life science research (see the BioSharing Information Resource), or the ARRIVE guidelines for reporting work involving animal research. Where applicable, authors should refer to any relevant reporting standards documents in this form.

If you have any questions, please consult our Journal Policies and/or contact us: editorial@elifesciences.org.

Sample-size estimation

- You should state whether an appropriate sample size was computed when the study was being designed
- You should state the statistical method of sample size computation and any required assumptions
- If no explicit power analysis was used, you should describe how you decided what sample (replicate) size (number) to use

Please outline where this information can be found within the submission (e.g., sections or figure legends), or explain why this information doesn't apply to your submission:

Explicit power analysis was not performed. Sample sizes were decided according to prior experience with analysing myelin using similar methods as in the present work, e.g., Patzig et al., eLIFE 2016; Patzig et al. Journal of Neuroscience 2011

Replicates

- You should report how often each experiment was performed
- You should include a definition of biological versus technical replication
- The data obtained should be provided and sufficient information should be provided to indicate the number of independent biological and/or technical replicates
- If you encountered any outliers, you should describe how these were handled
- Criteria for exclusion/inclusion of data should be clearly stated
- High-throughput sequence data should be uploaded before submission, with a private link for reviewers provided (these are available from both GEO and ArrayExpress)

Please outline where this information can be found within the submission (e.g., sections or figure legends), or explain why this information doesn't apply to your submission:

Biological replicates correspond to individual mice as indicated in the figure legends (n=...). Technical replicates apply exclusively to proteome analysis in Figure 1-source data 1, Figure 5 source data 1 and Figure 5 source data 2, and as detailed in the Materials and Methods section "Analysis of proteomic data ".

Statistical reporting

- Statistical analysis methods should be described and justified
- Raw data should be presented in figures whenever informative to do so (typically when N per group is less than 10)
- For each experiment, you should identify the statistical tests used, exact values of N, definitions of center, methods of multiple test correction, and dispersion and precision measures (e.g., mean, median, SD, SEM, confidence intervals; and, for the major substantive results, a measure of effect size (e.g., Pearson's r, Cohen's d)
- Report exact p-values wherever possible alongside the summary statistics and 95% confidence intervals. These should be reported for all key questions and not only when the p-value is less than 0.05.

Please outline where this information can be found within the submission (e.g., sections or figure legends), or explain why this information doesn't apply to your submission:

Statistical test and exact p-values for quantitative assessment of nerve sections are given in the Materials and Methods section "Histological analysis".
For proteome analysis, statistical methods are detailed in the Materials and Methods section "Analysis of proteomic data". Exact q-values are given in Figure 5-source data 2.

(For large datasets, or papers with a very large number of statistical tests, you may upload a single table file with tests, Ns, etc., with reference to sections in the manuscript.)

Group allocation

- Indicate how samples were allocated into experimental groups (in the case of clinical studies, please specify allocation to treatment method); if randomization was used, please also state if restricted randomization was applied
- Indicate if masking was used during group allocation, data collection and/or data analysis

Please outline where this information can be found within the submission (e.g., sections or figure legends), or explain why this information doesn't apply to your submission:

Experimental groups were allocated to groups dependent on genotype and age. Experimental mutant mice were analyzed together with either littermate controls or with wild type mice of the same age that were bred in the same mouse colony, as indicated in the respective Materials and Methods sections.

Additional data files ("source data")

- We encourage you to upload relevant additional data files, such as numerical data that are represented as a graph in a figure, or as a summary table
- Where provided, these should be in the most useful format, and they can be uploaded as "Source data" files linked to a main figure or table
- Include model definition files including the full list of parameters used
- Include code used for data analysis (e.g., R, MatLab)
- Avoid stating that data files are "available upon request"

Please indicate the figures or tables for which source data files have been provided:

Figure 1-source data 1. Label-free quantification of proteins in wild-type PNS myelin fractions by three different data acquisition modes
Figure 3-source data 1. Normalized mRNA abundance data
Figure 5-source data 1. Label-free quantification of proteins in PNS myelin fractions from Prx-/- mice by MSe
Figure 5-source data 2. Label-free quantification of proteins in PNS myelin fractions from WT and Prx-/- mice by DRE-UDMSe
